# Effect of mass ratio on micro-mesoporous Cu-SSZ-13/CeWTi composite catalysts for the selective catalytic reduction of NO with ammonia

**DOI:** 10.1039/d1ra03317d

**Published:** 2021-07-19

**Authors:** Wenyi Zhao, Menglin Shen, Yueran Zhu, Dongjie Wang, Xingang Li

**Affiliations:** Collaborative Innovation Center of Chemical Science and Engineering (Tianjin), Tianjin Key Laboratory of Applied Catalysis Science & Technology, School of Chemical Engineering & Technology, Tianjin University Tianjin 300350 China xingang_li@tju.edu.cn; National Key Laboratory for Research and Comprehensive Utilization of Rare Earth Resources in Baiyun Obo, Baotou Research Institute of Rare Earths Baotou 014030 China

## Abstract

A series of micro-mesoporous SSZ-13/Ce*x*W*y*Ti*z* composites with different zeolite/oxide ratios were synthesized using a one-step crystallization method. The effects of the mass ratio on the crystal form, specific surface area, pore structure, surface element properties, redox properties, surface acidity and deNO_*x*_ performance of the Cu-SSZ-13/CeWTi composite catalysts were investigated using X-ray diffractometry (XRD), Brunauer–Emmett–Teller analysis (BET), X-ray photoelectron spectroscopy (XPS), H_2_ temperature programmed reduction (H_2_-TPR) and NH_3_ temperature programmed desorption (NH_3_-TPD). The results reveal that the Cu-SSZ-13/CeWTi composite catalysts formed a micro-mesoporous structure. The increase in the mass ratio leads to the increased microporous ratio of the composite catalysts, the improved crystal structure of SSZ-13 and a higher specific surface area and pore volume, which is conducive to enhancing the low-temperature deNO_*x*_ activity, but its high-temperature performance (450 °C and above) decreases. The introduction of micropores into mesoporous materials can result in the production of more Ce^4+^, surface chemisorption oxygen species O_α_ and acid sites. The Cu-SSZ-13/CeWTi composite catalyst with a mass ratio of 1 : 4 demonstrated the best micro-mesoporous ratio, low-temperature selective catalytic reduction (SCR) performance and hydrothermal stability.

## Abstract 2

A series of micro-mesoporous SSZ-13/Ce*x*W*y*Ti*z* composites with different zeolite/oxide ratios were synthesized using a one-step crystallization method.

## Graphical abstract

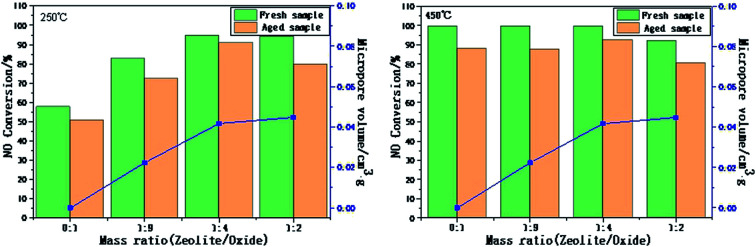

## 1. Introduction

Nitrogen oxide (NO_*x*_) is a key cause of photochemical smog, acid rain and the greenhouse effect. Generally, processes involving the combustion of fuel at high temperatures inevitably generate nitrogen oxides, which cannot be directly emitted without pre-treatment. NH_3_-selective catalytic reduction (SCR) technology is the prevailing denitration technology used at present, and its performance strongly depends on catalysts.

At present, the V_2_O_5_-WO_3_/TiO_2_ and Cu-SSZ-13 catalysts are commercially available. Vanadium based catalysts are widely used in flue gas denitration and diesel exhaust denitration owing to their good resistance to sulfur poisoning and the low production costs.^1–3^ However, issues such as the narrow activity temperature window, the volatilization of vanadium at high temperatures and secondary chemical pollution inhibit their further application.^4,5^ As an alternative to vanadium based catalysts, rare earth based catalysts, with the advantages of a low price and abundant resources, have attracted increasing attention. Among these metals, cerium, a light rare earth element with high natural reserves and a low price, has the potential to be applied in large-scale emission control units. CeO_2_ has a prominent oxygen storage and release capacity and is often combined with transition metal oxides in deNO_*x*_ catalysts.^6,7^ Cheng *et al.*^8^ reported that doping cerium into a TiO_2_ support increases the quantity of the surface adsorbed oxygen, thus improving the SCR activity. On the other hand, tungsten is often incorporated as an additive in cerium based catalysts for denitration. It was observed that the Ce–W–Ti catalyst prepared at pH = 9 exhibited an excellent SCR activity, and the NO_*x*_ conversion remained above 90% in the range of 300–450 °C.^9^ Cao Li *et al.*^10^ investigated the effect of the Ce/W molar ratio on the deNO_*x*_ performance of the CeO_2_–WO_3_/TiO_2_ honeycomb catalyst. It was found that the optimal Ce/W molar ratio was 4 : 1, and the NO_*x*_ conversion reached 73% at 275 °C and over 95% above 300 °C. However, both of these studies highlighted that the Ce–W-based catalysts are efficient at medium and high temperatures, but showed a relatively poor deNO_*x*_ performance at low temperatures. New emission regulations requiring an improved NO_*x*_ removal efficiency in diesel engines during a cold start and the emission requirements of furnaces in the non electric power industry have resulted in an urgent demand for low-temperature denitrification catalysts. Cu-SSZ-13 is a chabazite (CHA) type microporous molecular sieve, owing to its good low-temperature SCR performance and hydrothermal stability, Cu-SSZ-13 has become a mainstream catalyst that is used to meet the requirements of the China National six emission standards.^11,12^ Kwak *et al.*^13^ concluded that Cu-SSZ-13 retains its skeleton structure and showed an excellent activity after long-term hydrothermal aging treatment at high temperatures, while Cu-ZSM-5, Cu-Beta and Cu–Y were found to undergo collapse of the skeleton, a reduction in activity and even deactivation. Fan *et al.*^11^ reported that when the Si/Al ratio was 15, the catalyst contained sufficient isolated Cu^2+^ ions and Brønsted acid sites, and the conversion of NO reached 95% in the temperature range of 200–450 °C. Nevertheless, the high temperature performance above 500 °C was observed to be less than 80%. Therefore, in order to adapt to different working conditions, the preparation conditions of the Cu-SSZ-13 catalyst should be gradually adjusted and its high-temperature SCR performance can be improved to enable development to meet the demands of emission regulations in the future.

In this paper, a series of micro-mesoporous SSZ-13/Ce*x*W*y*Ti*z* composites with different mass ratios were prepared by combining the microporous molecular sieve SSZ-13 with the mesoporous material CeWTi. The micro-mesoporous Cu-SSZ-13/CeWTi composite catalyst was found to have the best micro-mesoporous ratio, low temperature SCR performance and hydrothermal stability and was developed by loading it with active components such as copper and cerium.

## 2. Experimental

### 2.1 Catalyst preparation

#### Synthesis of CeO_2_/WO_3_–TiO_2_

CeO_2_/WO_3_–TiO_2_ powder (denoted as Ce*x*W*y*Ti*z*; in which, *x* represents the weight percentage of CeO_2_, *x* = 20%; *y* represents the weight percentage of WO_3_, *y* = 8%; and *z* represents the weight percent of TiO_2_, *z* = 72%) were prepared using the impregnation method. Cerium nitrate (Ce(NO_3_)_3_·3H_2_O) and the TiO_2_–WO_3_ support were used for the experiment. The relative purity of cerium oxide in cerium nitrate produced by Baotou Xinyuan Rare Earth High Tech Materials Co., Ltd, China was 99.99%, and the weight ratio of the tungsten oxide in the TiO_2_–WO_3_ powder produced by Shanghai Meili Lian Chemical Trade Co., Ltd, China was 10%. Cerium nitrate was dissolved in deionized water. TiO_2_–WO_3_ power was added to the above solution and stirred for 1 h. The mixture was spray dried at 180 °C and calcined at 550 °C for 3 h in static air.

#### Configuration of the SSZ-13 precursor solution

The SSZ-13 molecular sieve precursor solution was prepared using the molar ratio of 2Na_2_O : Al_2_O_3_ : 30SiO_2_ : 10TMAdaOH : 900H_2_O. Sodium metaaluminate with a purity of ≥98% and sodium hydroxide with a purity of ≥96% were obtained from Sinopharm Chemical Reagent Co., Ltd, China. 1-Adamantyl trimethylammonium hydroxide solution (the solid content was 25%) was obtained from Kent Catalyst Materials Co., Ltd, China, and silica sol solution (SiO_2_·5H_2_O, the solid content was 30%, pH = 9–11) was purchased from Qingdao MAC Silica Gel Co., Ltd, China. The specific configuration process was performed as follows: firstly, a certain amount of NaAlO_2_ was dissolved in deionized water, and TMAdaOH was added slowly to the above solution. Secondly, after stirring evenly, the mixed solution was quickly added to a certain amount of NaOH and stirred for 30 min. Finally, the precursor solution was obtained by adding a silica sol solution (solid content is 30%, pH = 9–11) and the resulting solution was stirred for 2 h and aged at room temperature for 12 h.

#### Compounding of SSZ-13 and Ce*x*W*y*Ti*z*

Ce*x*W*y*Ti*z* powder with a certain mass ratio was added to a certain volume of the SSZ-13 precursor solution. After stirring, it was added into a hydrothermal reactor and crystallized at 180 °C for 24 h. The hydrothermal reactor is a stainless steel hydrothermal reactor with a PTFE lining. The crystallized samples were filtered and washed until the pH of the solution was neutral. Then, the Na type SSZ-13/Ce*x*W*y*Ti*z* composites were prepared by drying at 90 °C overnight and were then calcined at 550 °C for 8 h.

#### Preparation of H-SSZ-13/**Ce*x*W*y*Ti*z***

A certain amount of the Na type SSZ-13/Ce*x*W*y*Ti*z* composites were added into a 1.0 mol L^−1^ NH_4_NO_3_ solution according to the ratio of solid to liquid of 1 : 50 (g: mL), stirred in a water bath at 80 °C for 6 h and stirred and washed three times under the same exchange conditions. After washing and drying, NH_4_ type SSZ-13/Ce*x*W*y*Ti*z* powders were obtained, and then calcined at 550 °C for 8 h to prepare the H type SSZ-13/Ce*x*W*y*Ti*z* composites, in which the mass ratios of H-SSZ-13 to Ce*x*W*y*Ti*z* were 0 : 1, 1 : 9, 1 : 4 and 1 : 2, respectively.

#### Preparation of the Cu-SSZ-13/CeWTi composite catalysts

A certain amount of the H-SSZ-13/Ce*x*W*y*Ti*z* composites with different mass ratios were added into a 0.01 mol L^−1^ copper acetate solution slowly during stirring, and then ion exchanged in a water bath at 90 °C for 6 h, filtered, washed until the solution reached neutral, dried overnight at 90 °C and calcined at 550 °C for 8 h to obtain the Cu-SSZ-13/CeWTi composite catalysts with different mass ratios. The mass ratio represents the mass ratio of the H-SSZ-13 molecular sieve to Ce*x*W*y*Ti*z* oxide. The fresh catalyst was thermally aged in 10% H_2_O in air at 600 °C for 50 h and labeled as aged-600 °C-50 h. The durability of the catalyst was expressed using the deterioration rate, which is the average value for the decrease in NO conversion for the aged samples at four temperature test points (200, 250, 450 and 500 °C).

### 2.2 Evaluation of the catalytic activity

The activity of 0.06 g of the catalysts was tested in a fixed-bed quartz reactor (inner diameter = 6 mm). The gas mixture simulates a real diesel exhaust, which contains 200 ppm NO, 200 ppm NH_3_, 200 ppm CO, 4.5 vol% CO_2_, 50 ppm C_3_H_6_, 12 vol% O_2_, 5 vol% H_2_O and N_2_ as the balance gas. The total flow rate was 300 mL min^−1^, corresponding to a gas hourly space velocity (GHSV) of 300 000 h^−1^. The effluent gas, including NO, NO_2_, and O_2_ was continuously analyzed using an online flue gas analyzer. The results for the steady-state activity were collected after 20 min at each temperature. The NO conversion was calculated as follows:
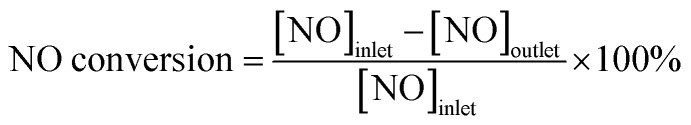


### 2.3 Characterization of the catalyst

X-ray powder diffractometry (XRD) analysis was performed on a panalytical X-ray powder diffraction analyzer. A Cu Kα target (*λ* = 1.5406 Å) was used as the radiation source. The test conditions used were a tube current of 40 mA and a tube voltage of 40 kV. During the test, wide-angle XRD scanning was performed at a speed of 2° min^−1^ in the range of 2*θ* = 5–90° with a step size of 0.02°.

The Brunauer–Emmett–Teller analysis (BET) and pore structure distribution were measured at −196 °C on a 3H-2000PM2 physical adsorption instrument manufactured by the Bester company by using the nitrogen adsorption–desorption method. The surface area and pore size distribution curve were determined using the density functional theory (DFT) method in the 0–0.3 partial pressure range.

X-ray photoelectron spectroscopy (XPS) analysis was obtained using a Thermo ESCALAB 250Xi spectrometer (ThermoFisher Scientific, Waltham, MA, USA) with Al Ka radiation (1486.6 eV). The binding energy (B.E.) spectrum was calibrated according to the C 1s standard spectrum (B.E. = 284.6 eV). The composition on surface of the catalyst according to the atomic ratios was calculated, and the Shirley background and Gaussian–Lorentzian methods were used for peak analysis.

H_2_ temperature programmed reduction (H_2_-TPR) was measured using a Quantachrome: Chem BET chemisorption analyzer (Micromeritics, Norcross, GA, USA) and a thermal conductivity detector (TCD) detector was used. Before the test, 50 mg of the sample was heated from room temperature to 800 °C at a rate of 10 °C min^−1^. A mixed gas flow of 5 vol% H_2_/N_2_ was used as the reductant at a flow rate of 60 mL min^−1^. The gas was purified using a trap containing CaO + NaOH materials in order to remove the H_2_O and CO_2_.

NH_3_ temperature programmed desorption (NH_3_-TPD) was conducted on a Quantachrome: Chem BET chemisorption analyzer (Micromeritics, Norcross, GA, USA). 50 mg of the sample was pretreated at 300 °C in an N_2_ flow at 20 mL min^−1^ for 2 h, then cooled down to 40 °C. It was adsorbed in 5 vol% NH_3_/N_2_ for 30 min (20 mL min^−1^). After that, the NH_3_ was turned off, the gas flow was switched back to pure N_2_ (20 mL min^−1^) again and it was purged for 30 min to remove the physically adsorbed species. Then, the desorption was completed by increasing the temperature from 40 to 800 °C at a rate of 10 °C min^−1^.

## 3. Results and discussion

### 3.1 SCR activity

**Fig. 1:**
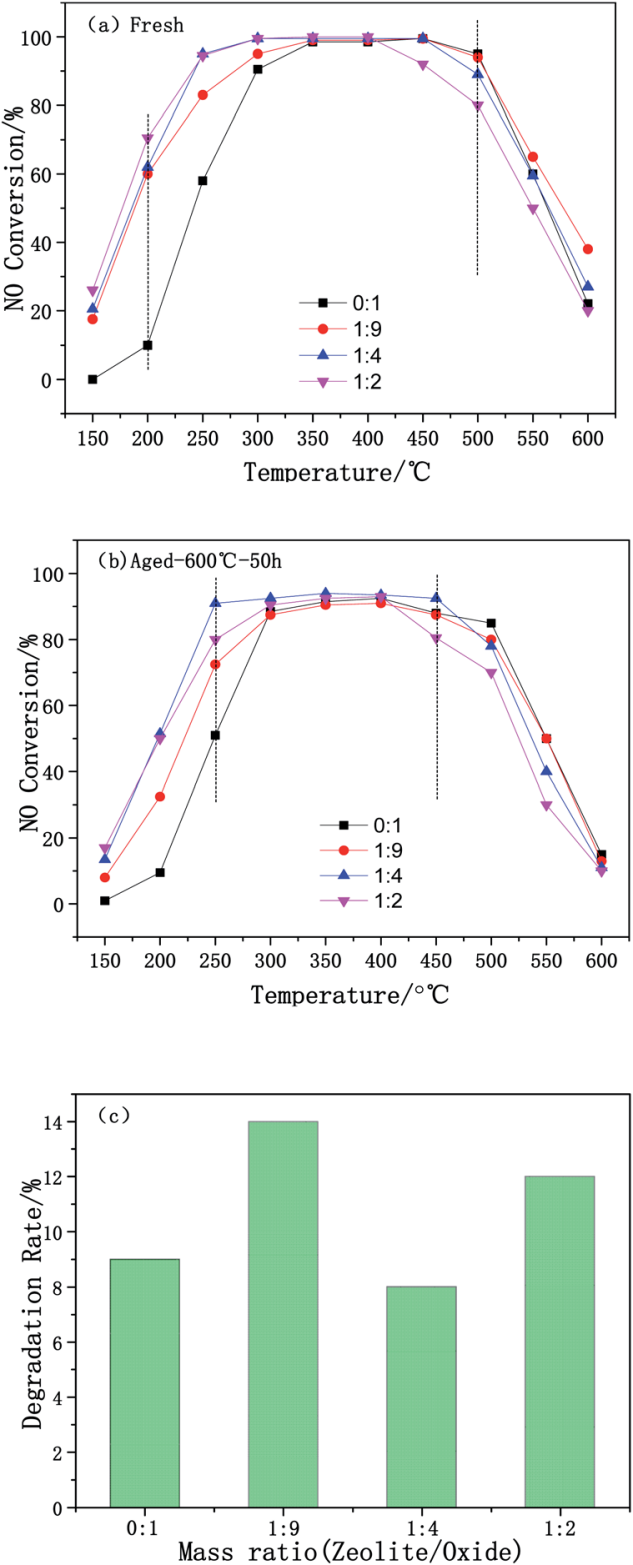
DeNO_*x*_ performance of the Cu-SSZ-13/CeWTi composite catalysts with different mass ratios: (a) fresh samples; (b) hydrothermally aged samples; and (c) degradation rate.

The effect of the mass ratio on the deNO_*x*_ performance of the Cu-SSZ-13/CeWTi composite catalysts is illustrated in Fig. 1. As shown in Fig. 1a, with the increase in the mass ratio, the catalytic activity of the composite catalysts at 150–300 °C was significantly improved and the reaction window was effectively widened, indicating that adding micropores can effectively promote the deNO_*x*_ performance of the catalysts at low temperatures.^14^ The pure CeWTi catalyst shows a relatively low catalytic activity in the low-temperature region of 150–250 °C. The NO_*x*_ conversion only reaches 10% at 200 °C, but this value increases to more than 95% at 350–500 °C. The catalytic activity of the Cu-SSZ-13/CeWTi composite catalysts with mass ratios of 1 : 4 and 1 : 2 were relatively high at 150–400 °C, and the NO_*x*_ conversion increased to more than 60% at 200 °C. Nevertheless, the NO_*x*_ conversion at 450 °C and above for composite catalysts with a mass ratio of 1 : 2 is significantly lower than that of the other catalysts.

The SCR performance of the catalysts after the hydrothermal aging treatment is shown in Fig. 1b. The durability and stability of the catalyst are expressed by the deterioration rate, and this is shown in Fig. 1c. Compared with the fresh samples, the smaller the degradation rate of the aged catalyst, the higher its stability and durability. In Fig. 1c, the degradation rate of aged samples does not change significantly with the increase of the mass ratio. The degradation rate was found to be only 8% for aged samples with a mass ratio of 1 : 4 and the NO_*x*_ conversion, especially at 250 °C still reached more than 90% after aging, suggesting that the composite catalyst with a mass ratio of 1 : 4 has a superior hydrothermal stability.

**Fig. 2:**
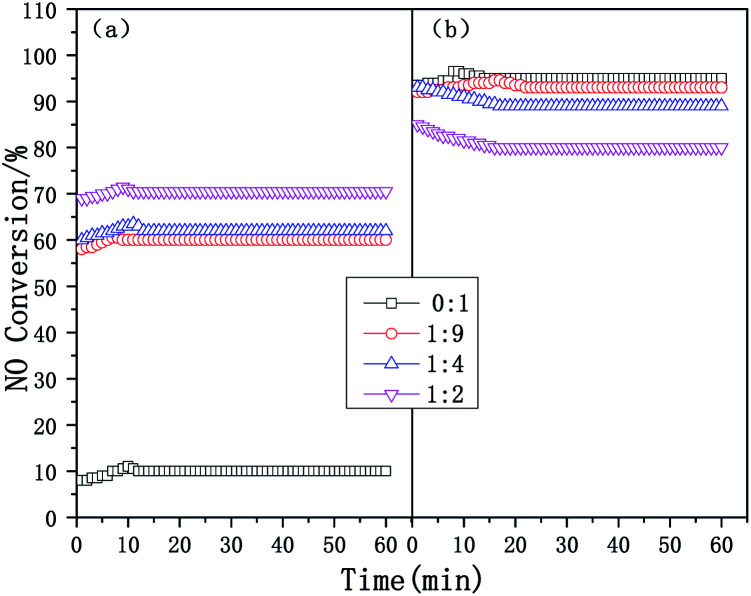
The time-on-stream behaviors of the Cu-SSZ-13/CeWTi composite catalysts with different mass ratios in the SCR reaction: (a) 200 °C and (b) 500 °C.

Fig. 2 exhibits the time-on-stream behaviors of the composite catalysts at 200 and 500 °C. At different temperatures, the NO_*x*_ conversion of the composite catalysts tends to be stable after 10–20 min, therefore it is only necessary to compare the NO_*x*_ conversion of the catalysts after 20 min. When the reaction time increased from 20 to 60 min, the SCR performance of the catalysts remained stable. The NO_*x*_ conversion of the composite catalysts at 200 °C increases significantly with an increase in the mass ratio, but the NO_*x*_ conversion of the composite catalysts at 500 °C shows the opposite trend, it is further shown that adding micropores is beneficial and improves the low-temperature SCR performance (300 °C and below), but the high-temperature performance (450 °C and above) of the composite catalyst decreases.

### 3.2 XRD analysis

**Fig. 3:**
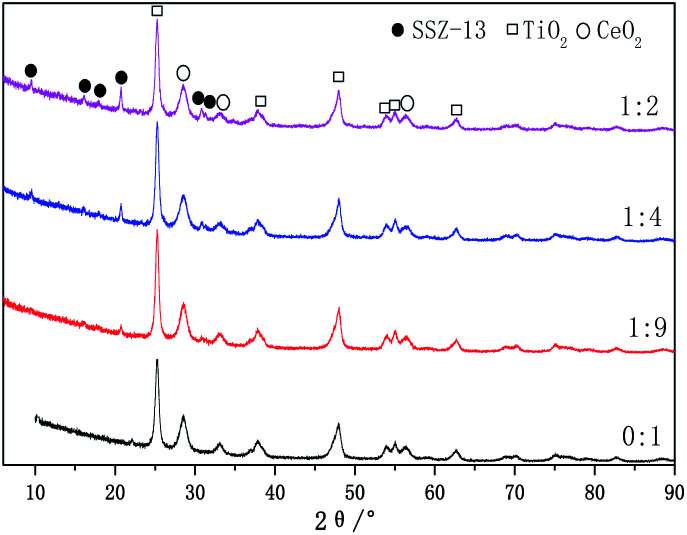
XRD patterns of the Cu-SSZ-13/CeWTi composite catalysts with different mass ratios.

The XRD patterns of the Cu-SSZ-13/CeWTi composite catalysts are presented in Fig. 3. It can be seen that the diffraction peaks of the anatase structure TiO_2_ (ICDD PDF# 21-1272) and the cubic fluorite structure CeO_2_ (ICDD PDF#34-0394) can be detected in the Cu-SSZ-13/CeWTi composite catalysts at different mass ratios, but no WO_3_ or CuO phase is observed, which suggests that the W species may exist in the amorphous form or as a highly dispersed species,^15,16^ while the Cu species is stable in the microporous structure of the SSZ-13 molecular sieves in the form of Cu^2+^.^17^ In addition to the obvious diffraction peaks of TiO_2_ and CeO_2_ in the Cu-SSZ-13/CeWTi composite catalysts with mass ratios of 1 : 9, 1 : 4 and 1 : 2, there are also six characteristic peaks of SSZ-13 in the range of 8–22°and 30–32°. The intensity of the SSZ-13 peaks increases remarkably with the increasing mass ratio, indicating the higher degree of crystallinity of the SSZ-13 phase. This may be due to the decrease in the amount of Ce*x*WyTi*z* powder in the SSZ-13 precursor solution, which can effectively improve the growth rate of the SSZ-13 crystal and is more conducive to the nucleation of the molecular sieve. When the mass ratios are 1 : 4 and 1 : 2, the Cu-SSZ-13/CeWTi composite catalysts can be synthesized with a better crystal structure of SSZ-13.

**Fig. 4:**
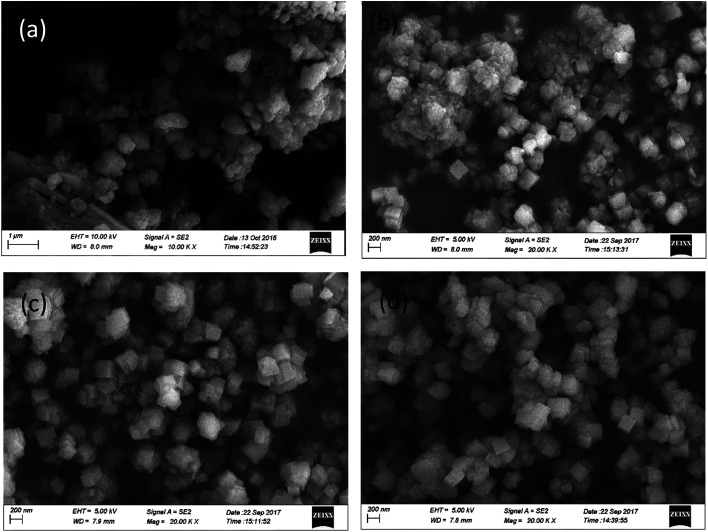
SEM images of the Cu-SSZ-13/CeWTi composite catalysts with different mass ratios: (a) 0 : 1; (b) 1 : 9; (c) 1 : 4; and (d) 1 : 2.

The SEM images of the catalysts with different mass ratios are exhibited in Fig. 4. As shown in Fig. 4a–d, prior to the addition of SSZ-13, the pure CeWTi catalyst exhibits an irregular particle structure and the particles adhere to each other. When the mass ratio of the Cu-SSZ-13/CeWTi composite catalysts increases from 1 : 9 to 1 : 2, the morphological structure gradually changes from a large number of irregular aggregates and a small number of cube structures to a more complete cubic structure, indicating that the CHA structure in the composite catalyst has gradually improved, which is consistent with the XRD results.

### 3.3 BET and pore structure analysis

**Fig. 5:**
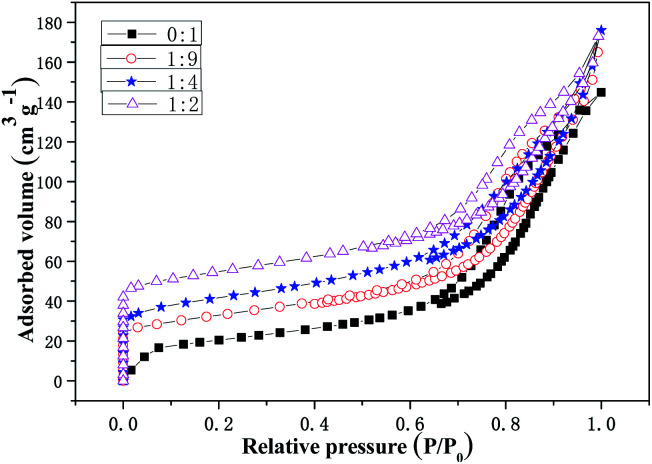
N_2_ adsorption–desorption isotherms of the Cu-SSZ-13/CeWTi composite catalysts with different mass ratios.

The N_2_ adsorption–desorption isotherms of the Cu-SSZ-13/CeWTi composite catalysts with mass ratios of 0 : 1, 1 : 9, 1 : 4 and 1 : 2 are shown in Fig. 5. The pore structure parameters of the composite catalysts are listed in Table 1. As shown in Fig. 5, the increase in the mass ratio results in an increase in the adsorption capacity. As shown in Table 1, the specific surface area and total pore volume of the Cu-SSZ-13/CeWTi composite catalysts also increased, and the average pore size decreased. The results show that the adsorption–desorption isotherm for the CeWTi catalyst with a mass ratio of 0 : 1 is type IV, and a H1 type hysteresis loop appears in the relative pressure *P*/*P*_0_ range of 0.6–1.0, which indicates that the CeWTi catalyst is a cylindrical homogeneous mesoporous material with a narrow pore size distribution.^14^ If *P*/*P*_0_ is close to 1, the adsorption–desorption isotherm for the CeWTi catalyst shows a saturated adsorption platform, which illustrates that the CeWTi catalyst is a mesoporous material with a single ordered pore arrangement. The adsorption–desorption isotherms for the Cu-SSZ-13/CeWTi composite catalysts synthesized at mass ratios of 1 : 9, 1 : 4 and 1 : 2 are also type IV, indicating the existence of the mesopore.^18,19^ H4 type hysteresis loops are found for the relative pressure range *P*/*P*_0_ of 0.6–1.0. H4 type hysteresis loops are generally discovered in the aggregated crystals of the zeolites, some mesoporous zeolites and micro-mesoporous carbon materials.^14^ Although the relative pressure *P*/*P*_0_ is less than 0.6, there was a very obvious adsorption capacity, which is related to the micropore filling, indicating that composite catalysts with mass ratios of 1 : 9, 1 : 4 and 1 : 2 formed the micro-mesoporous structure. If *P*/*P*_0_ is close to 1, there is no saturated adsorption platform in the composite catalysts, which suggests that there is not a single ordered pore arrangement, which further proves that the composites are micro-mesoporous materials.

**Table 1:** Pore structure parameters of the Cu-SSZ-13/CeWTi composite catalysts with different mass ratios

Mass ratio	*S*_BET_/m^2^ g	Total pore volume/cm^3^ g	Average pore size/nm	*S*_BET_/m^2^ g		Pore volume/cm^3^ g	
				Mesoporous	Microporous	Mesoporous	Microporous
0 : 1	71.8	0.2240	12.47	71.8	0	0.2240	0
1 : 9	113.5	0.2551	8.99	60.5	40.5	0.2200	0.0224
1 : 4	146.1	0.2622	7.45	50.6	86.4	0.2160	0.0418
1 : 2	179.4	0.2676	5.37	45.4	95.6	0.1896	0.0448

**Fig. 6:**
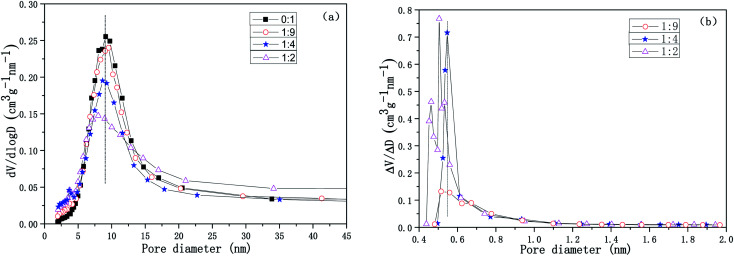
Pore size distribution curves for the Cu-SSZ-13/CeWTi composite catalysts with different mass ratios. (a) Mesoporous pore size distribution curve; and (b) microporous pore size distribution curve.

The pore size distribution curves for the Cu-SSZ-13/CeWTi composite catalysts with different mass ratios are shown in Fig. 6. It is clear that with the increasing mass ratio, the mesoporous volume of the composite catalysts decreases gradually, while the microporous volume increases significantly, which is consistent with the results shown in Table 1. If the mass ratio increases from 0 : 1 to 1 : 4, the average pore diameter is approximately 9 nm with a narrow mesoporous pore size distribution and a high degree of order. However, if the mass ratio continues to increase to 1 : 2, the average pore diameter decreases to 7.5 nm, corresponding to an increase in the specific surface area and the total pore volume of the composite catalyst, but the mesoporous pore size distribution is wider, and the degree of order is lower. According to the microporous pore size distribution and the results shown in Table 1, when the mass ratio increases from 1 : 9 to 1 : 4, the peak of the microporous pore size distribution curve is more obvious. Its distribution becomes narrower and the degree of order is improved, and the microporous volume increases remarkably from 0.0224 to 0.0418 cm^3^ g. If the mass ratio is increased further to 1 : 2, the microporous pore size distribution of the composite catalyst widens and also splits into two peaks. Meanwhile, the degree of order decreased and the rate of increase in the micropore volume was reduced, to only 0.0448 cm^3^ g. Hence, the microporous pore size distribution of the composite catalyst with mass ratio of 1 : 4 was the narrowest and the degree of order reached the highest volume.

**Fig. 7:**
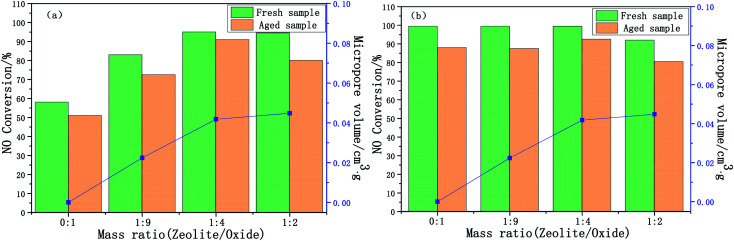
Relationship between the SCR activity and the micropore volume for the Cu-SSZ-13/CeWTi composite catalysts with different mass ratios at 250 °C (a) and 450 °C (b) before and after hydrothermal aging.

Fig. 7 shows the relationship between the SCR activity and the micropore volume of the catalysts with different mass ratios, the higher micropore ratio can be observed in the composite catalysts, the larger the specific surface area and pore volume, the more favorable the contact between the active center and the reactant molecules, which results in an improved catalytic activity, especially for the performance of the SCR at low temperature.^20^ Whereas the over high proportion of micropores in the composite catalysts leads to an inhibited high-temperature SCR performance (above 450 °C), which may be due to the formation of a large amount of CuO*x* precipitating from the micropore channel after Cu^2+^ in the CHA cage is oxidized at high temperatures, resulting in the destruction of the structure of the CHA cage and the micropores.^21^ Consequently, the Cu-SSZ-13/CeWTi composite catalyst with a mass ratio of 1 : 4 showed the optimum micro-mesoporous ratio, a low temperature SCR performance and hydrothermal stability.

### 3.4 XPS analysis

**Fig. 8:**
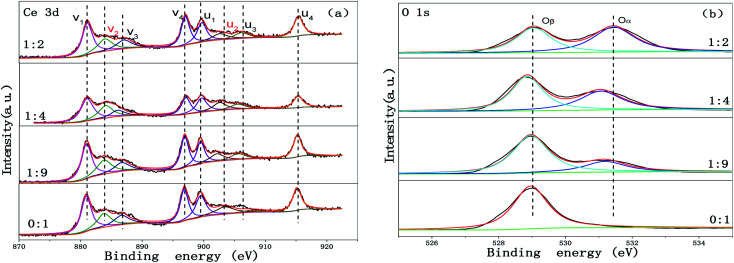
XPS spectra of the Cu-SSZ-13/CeWTi composite catalysts with different mass ratios: (a) XPS spectrum of Ce 3d, and (b) XPS spectrum of O 1s.

In order to understand the chemical states of the elements over the surface of the Cu-SSZ-13/CeWTi composite catalysts with different mass ratios, the XPS spectra for Ce and O are presented in Fig. 8. The surface element concentrations of the composite catalysts are summarized in Table 2. It can be seen from Table 2 that with the increase in the mass ratio, and the contents of Ce, W and Ti on the surface of the composite catalysts decrease gradually, while the surface contents of Cu and O increase significantly, indicating that a greater amount of microporous materials is exposed on the catalyst surface. In Fig. 8a, eight peaks in the Ce 3d XPS spectra of the composite catalysts are observed, which can be divided into two u and v series, corresponding to the Ce 3d_3/2_ and 3d_2/5_ orbit, respectively. According to the previously published literature,^6,9^ u_2_ and v_2_ can be classified as the characteristic peaks of Ce^3+^, while u_1_, u_3_, u_4_, v_1_, v_3_ and v_4_ were ascribed as the characteristic peaks of Ce^4+^. The ratio of Ce^4+^/(Ce^3+^ + Ce^4+^) was calculated according to the deconvoluted peak areas and used to measure the concentration of the Ce^4+^ species on the surface of the catalyst. According to the area ratio of the Ce peaks, the surface Ce^4+^ concentrations of the Cu-SSZ-13/CeWTi composite catalysts with mass ratios of 0 : 1, 1 : 9, 1 : 4 and 1 : 2 were 0.79, 0.79, 0.94 and 0.81, respectively. It can be seen that with the increase in the mass ratio, the Ce^4+^ concentration on the surface of the catalysts increases first and then decreases. This suggests that increasing the proportion of micropores in the composite catalysts is conducive to the increase in Ce^4+^ content on the surface of the catalysts, but with a further increase in the proportion of the micropores, this growth is inhibited to a certain extent. Many studies have confirmed that the existence of Ce^4+^ and its redox process may be the main reason for the conversion of the NH_3_ that adsorbs onto the surface of the catalyst into the intermediate product NH_2_, which can react with gaseous NO to form N_2_ and H_2_O, thus enhancing the SCR activities.^22,23^

**Table 2:** Surface element properties of the Cu-SSZ-13/CeWTi composite catalysts with different mass ratios

Mass ratio	Surface element concentration/at%					Ce^4+^/(Ce^3+^ + Ce^4+^)	O_α_/(O_α_ + O_β_)
	Ce	W	Ti	O	Cu		
0 : 1	3.60	2.53	22.02	57.01	0	0.79	0
1 : 9	3.44	2.00	17.25	60.25	0.18	0.79	0.25
1 : 4	2.07	1.71	15.36	61.19	0.87	0.94	0.42
1 : 2	2.64	1.42	12.78	67.91	0.99	0.81	0.54

Fig. 8b displays the O 1s XPS spectra of the catalysts with different mass ratios. The O 1s spectra can be fitted into two peaks, which are assigned to the lattice oxygen species (labeled as O_β_) at lower binding energies (528.8–529.1 eV) and the surface chemisorption oxygen species (labeled as O_α_) at higher binding energies (531.3–531.9 eV). In this paper, the ratio of O_α_/(O_α_ + O_β_) is used to measure the concentration of the chemisorbed oxygen species on the surface of the catalyst. As shown in Fig. 8b and Table 2, the peak intensity of the surface chemisorption oxygen O_α_ was dramatically increased as the mass ratio increased, and the peak intensity of the lattice oxygen species O_β_ decreased, then the ratio of O_α_/(O_α_ + O_β_) was notably elevated from 0 to 0.54. The surface adsorbed oxygen is considered to be more reactive in oxidation reactions because the higher mobility is comparable to that of lattice oxygen,^18,24^ and a significant amount of research shows that a high O_α_/O_β_ ratio could enhance the oxidation of NO to NO_2_ in the SCR reaction, which favors the “fast SCR” reaction.^25^ The XPS results are consistent with the activity test results shown in Fig. 1a.

### 3.5 Redox properties (H_2_-TPR)

**Fig. 9:**
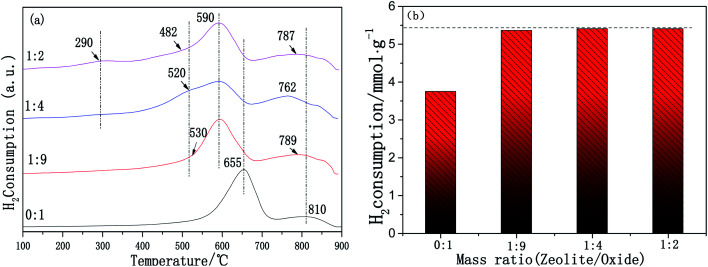
H_2_-TPR patterns for the Cu-SSZ-13/CeWTi composite catalysts with different mass ratios: (a) H_2_-TPR patterns; and (b) H_2_ consumption.

It is generally accepted that the redox properties are an important factor affecting the NH_3_-SCR reaction. The redox properties of the samples were characterized using H_2_-TPR. The H_2_-TPR patterns of the Cu-SSZ-13/CeWTi composite catalysts with different mass ratios are shown in Fig. 9a. According to the literature,^26,27^ the low temperature reduction peak at about 290 °C can be attributed to the reduction peak of Cu^2+^ to Cu^+^, which only appears for the catalyst with a mass ratio of 1 : 2. The reduction peak of 482–530 °C is attributed to the reduction of Cu^+^ to Cu^0^. The location and area of this reduction peak are more dependent on the mass ratio and the pure CeWTi catalyst does not possess this reduction peak. With the increase in the mass ratio, the reduction temperature of Cu^+^ to Cu^0^ in the composite catalysts decreases gradually. This clearly shows that the reduction of Cu^+^ to Cu^0^ was easier at a higher mass ratio, probably owing to the increase in the micropore proportion in the composite catalysts, which increases the concentration of Cu^2+^ in the single eight ring window, resulting in the instability of the Cu^+^ species.^17^ According to previously published reports,^16^ the reduction peaks of pure CeO_2_ are 300–550 °C and 750–850 °C, corresponding to the reduction of the surface oxygen and the reduction of the bulk oxygen of CeO_2_, respectively. The reduction peaks of WO_3_ at 300–600 °C and 700–800 °C are attributed to the reduction of W^6+^ to W^4+^ and the reduction of W^4+^ to W^0^. In addition, the interaction between the CeO_2_ and WO_3_ also exists in the composite catalyst. Thus, there are two overlapping reduction peaks for CeO_2_ and WO_3_ at 590–655 °C and 762–810 °C. The first overlapping peaks range from 590 to 655 °C and are attributed to the reduction of W^6+^ to W^4+^, as well as the surface oxygen of CeO_2_. The second overlapping peaks between 762 and 810 °C are ascribed to the reduction of W^4+^ to W^0^, as well as the bulk oxygen of CeO_2_, revealing a strong interaction between the CeO_2_ and WO_3_ species.^28^ For the Cu-SSZ-13/CeWTi composite catalysts, the two overlapping reduction peaks of CeO_2_ and WO_3_ move to a lower temperature with the increasing mass ratio, which indicates that the reduction ability of the CeWTi mesoporous material in the composite catalysts is also significantly improved. To a certain extent, the addition of micropores weakens the interaction between CeO_2_ and WO_3_. With the increasing mass ratio, the consumption of H_2_ increases from 3.75 to 5.41 mmol g^−1^ and the concentration of the surface chemisorbed oxygen species also increases from 0 to 0.54 as shown in Fig. 9b and 8b. This indicates that with the increased proportion of micropores in the composite catalysts, a greater number of reducible subsurface oxygen sites are introduced that improve the redox performance, facilitating the SCR reaction. These results are consistent with the SCR activity.

### 3.6 Surface acidity (NH_3_-TPD)

**Fig. 10:**
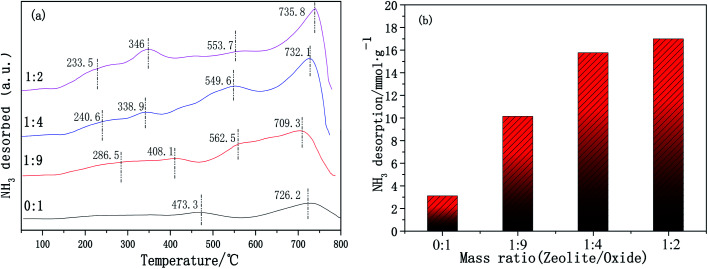
NH_3_-TPD patterns of the Cu-SSZ-13/CeWTi composite catalysts with different mass ratios: (a) NH_3_-TPD profiles; and (b) amount of NH_3_ desorption.

The surface acidity of the catalyst is another important factor affecting the catalytic performance for NH_3_-SCR. Surface acid sites promote ammonia adsorption on the catalyst surface, and the type and quantity of acid sites will affect the catalytic activity. The surface acidity of the Cu-SSZ-13/CeWTi composite catalysts with different mass ratios was tested using NH_3_-TPD and is shown in Fig. 10a. The CeWTi catalyst exhibits two desorption peaks in the temperature range of 100–800 °C with a mass ratio of 0 : 1. However, when the mass ratio of the Cu-SSZ-13/CeWTi composite catalysts increases from 1 : 9 to 1 : 2, there are four desorption peaks in the temperature range, which are attributed to the NH_3_ desorbed by weak (200–300 °C), medium strong (330–480) and strong (550–750) acid sites.^25^ Upon increasing the mass ratio from 0 : 1 to 1 : 2, the amount of NH_3_ desorption increases significantly from 3.09 to 16.99 mmol g^−1^, as seen in Fig. 10b. All these results imply that the addition of micropores can increase the amount of acid sites on the catalysts, resulting in a greater number of NH_3_ adsorption sites, which play an important role in the SCR reaction.^29^

## 4. Conclusion

In this paper, the effect of the mass ratio on micro-mesoporous Cu-SSZ-13/CeWTi composite catalysts is studied. The increase in the mass ratio can significantly improve the low temperature SCR performance of the catalyst and the optimal mass ratio was found to be 1 : 4. After the hydrothermal aging treatment, the degradation rate was only 8% and the catalytic activity could still reach more than 90% after aging at 250 °C, showing an excellent hydrothermal stability. The XRD results show that there was a decreased amount of Ce*x*WyTi*z* powder in the SSZ-13 precursor solution, which can effectively improve the growth rate of the SSZ-13 crystal and is more conducive to the nucleation of the molecular sieve. The BET and pore structure analysis results indicate that the higher the proportion of micropores in the composite catalyst, the larger the specific surface area and pore volume, meaning the catalyst is more conducive to contact between the active center and the reactant molecules. Therefore, the low-temperature deNO_*x*_ activity is improved, however, the high-temperature performance (450 °C and above) of the composite catalyst decreases. The XPS results prove that micropores can accelerate the electron transfer and oxygen transfer between the zeolite and CeWTi, increasing the Ce^4+^ content and the chemisorbed oxygen species on the surface of the catalyst, which is beneficial to improving the activity of the CeWTi component in the composite catalyst and further enhancing the SCR deNO_*x*_ performance. The H_2_-TPR results suggest that the addition of micropores can introduce subsurface oxygen species that are more easily reduced and improve the redox performance. The NH_3_-TPD results indicate that the micropores provide a greater number of acid sites and more adsorbed NH_3_ species, which is also beneficial to the SCR reaction.

## Author contributions

Devising and writing articles, Wenyi Zhao; organizing and designing experiments, Menglin Shen; experimental studies, Yueran Zhu; literature search and data sorting, Dongjie Wang; experimental guidance and revision of article, Xingang Li.

## Conflicts of interest

There are no conflicts to declare.

## Supplementary Material
